# Depletion-Induced Self-Assembly of Colloidal Particles on a Solid Substrate

**DOI:** 10.1021/acs.langmuir.4c00186

**Published:** 2024-04-11

**Authors:** Gideon Onuh, Daniel Harries, Ofer Manor

**Affiliations:** † The Wolfson Department of Chemical Engineering, Technion-Israel Institute of Technology, Haifa 3200000, Israel; ‡ The Fritz Haber Research Center, and the Harvey M. Kruger Center for Nanoscience & Nanotechnology, Institute of Chemistry, The Hebrew University, Jerusalem 9190401, Israel

## Abstract

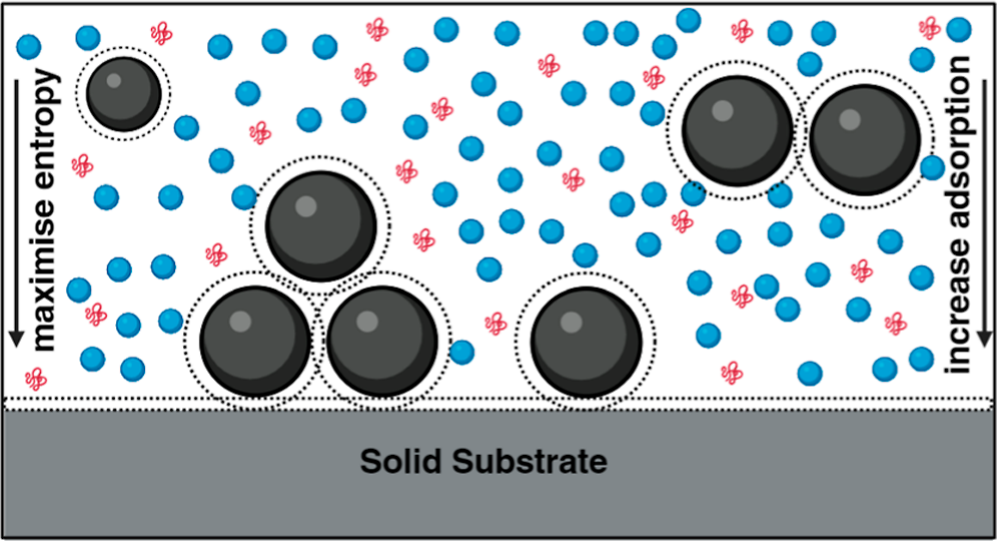

We investigate the depletion contributions to the self-assembly of microcolloids on solid substrates. The assembly is driven by the exclusion of nanoparticles and nonadsorbing polymers from the depletion zone between the microcolloids in the liquid and the underlying substrate. The model system consists of 1 μm polystyrene particles that we deposit on a flat glass slab in an electrolyte solution. Using polystyrene nanoparticles and poly(acrylic acid) polymers as depleting agents, we demonstrate in our experiments that nanoparticle concentrations of 0.5% (w/v) support well-ordered packing of microcolloids on glass, while the presence of polymers leads to irregular aggregate deposition structures. A mixture of nanoparticles and polymers enhances the formation of colloidal aggregate and particulate surface coverage compared to using the polymers alone as a depletion agent. Moreover, tuning the polymer ionization state from pH 4 to 9 modifies the polymer conformational state and radius of gyration, which in turn alters the microcolloid deposition from compact multilayers to flocculated structures. Our study provides entropic strategies for manipulating particulate assembly on substrates from dispersed to continuous coatings.

## Introduction

Depletion-induced self-assembly of macromolecules and particles has been the subject of extensive research due to its wide range of practical applications.^1−5^ The depletion force has been exploited to trigger the self-assembly of colloids, including concave and nonpatchy anisotropic nanoparticles with large flat facets and patchy colloids.^6−9^ For example, Rossi et al.,^10^ utilized depletion interactions to create photonic crystals with tunable optical properties. Another study by Baranov et al.,^11^ reported the fabrication of 2D close-packed hexagonal arrays using a depletion-induced assembly of nanorods of semiconductors and magnetic particles, demonstrating that the product exhibits photonic band gaps. Moreover, regioselective functionalization of colloidal particles was achieved through depletion-induced self-assembly, allowing for the creation of materials with tailored chemical and physical properties.^12^ In different types of applications, the preferential exclusion of nanoparticles or polymers from the vicinity of macromolecules in a crowded system generates a depletion zone that leads to an attractive depletion force.^13−15^ This may lead to so-called depletion flocculation and has been utilized in various fields such as water purification,^16−18^ virus particle assembly,^19,20^ and also underlies macromolecular crowding in biological cells.^21,22^

Despite the entropic origin of depletion forces, they are well-known to contribute to the assembly of particles and macromolecules.^14,23−26^ The preferential exclusion of depletants results in an osmotic pressure gradient between the pure solvent molecules in the intervening and excluded volumes, thereby generating attractive interactions between colloids.^27−29^ The strength of the interactions is influenced by various factors, in particular by the concentration and size of depletants.^30−32^ Minton^33^ develops an analytical model exploring how variation in concentration, aggregate shape, excluded volume effects, and surface adsorption influence the self-association and distribution of macromolecules. The model shows that depletion interactions arising from crowding, combined with surface adsorption, have a strongly cooperative effect in driving self-assembly and preferential adsorption of macromolecules on surfaces.

Few studies have explored the contribution of depletion to the assembly of these entities on solid surfaces. The assembly of particles on a substrate suggests practical implications in the development of new materials with unique properties,^34,35^ engineering thin films,^36,37^ and designing surfaces with specific functionalities.^38,39^ The self-assembly of microcolloids shows a close connection to colloidal interactions and surface forces between the colloids themselves and between colloids and substrates.^40−45^ Surface forces translate to particle coagulation in the bulk of the carrier liquid and particle adsorption to the solid substrate, two mechanisms that determine the substrate coverage level and morphology of the microcolloids deposit.^46−50^

Here, we explore the depletion-induced self-assembly of microcolloids on a solid substrate. We investigate the contribution of nanoparticles and polymers as depletants on the assembly morphology and substrate coverage of microcolloids at different concentrations and pH. We detail our experiments in Experiment, present our findings and assessment in Results and Discussion, and synthesize our findings in Conclusion.

## Experiment

### Sample Preparation

Polystyrene particles, 1 μm in diameter (cat. no. 89904) and poly(acrylic acid) (PAA) solution (MW: 100,000 Da) (cat. no. 416002) were purchased from Sigma-Aldrich, Rehovot, Israel. Polystyrene nanoparticles of 85 nm in diameter (cat. no. PP-008-100) were purchased from Spherotech, Inc., USA. The particles were stabilized in an aqueous suspension by anionic sulfate groups during the fabrication process, thereby enhancing their negative charge density. We prepared a 0.01% (w/v) micropolystyrene suspension in HPLC water and added different concentrations of nanoparticles [0.01, 0.05, 0.1, and 0.5% (w/v)] or PAA [0.01, 0.05, 0.1, and 0.5% (w/v)] to induce depletion interaction. To isolate the contribution of depletion attraction between microcolloids, we kept the ionic concentration at 5 mM NaNO_3_ (Spectrum, Sigma-Aldrich, 99%) throughout the experiments. We further manipulated the pH-responsive PAA polymer by adjusting the pH of the suspension from acidic to basic (4–9) using HCl [ACS reagent, Sigma-Aldrich, 37% (w/v)] and NaOH (spectrum, Sigma-Aldrich, 98%). The pH of the suspension was monitored using the Thermo Scientific Eutech pH 2700 pH Meter during the stepwise addition of small volumes (<0.1 mL) of 0.1 M HCl and NaOH under constant stirring. We chose pH 4–9 as it spans below and above the polymer’s known p*K*_a_ of approximately 4.5,^51^ enabling changes to its carboxylate ionization and conformational structure.^52,53^

### Particle Deposition

In our experiments, we monitor the deposition of particles on a standard microscope glass slide (3.5 × 2 mm^2^, Sigma-Aldrich, 0.1 μm thickness). The glass slide was cleaned with acetone (GADOT, 99.8%), isopropyl alcohol (GADOT, 96% pure), ethanol (GADOT, 96 pure), and deionized water (Macron, HPLC grade) to remove surface contamination. We treated the glass slide with oxygen plasma for 10 min to generate hydrophilic functional groups on the surface and heated it at 100 °C for 30 min. We placed the substrate in a closed glass Petri dish (BR455751, Sigma-Aldrich, 4.5 mm in diameter, 1 mm in height) filled with the prepared suspension and monitored the deposition for 4 days. The deposition reached a steady state after 4 days; from this time point, we observed no significant change in the particulate deposition.

### Instrumentation

The morphology and structure of the self-assembled pattern aggregate structures were analyzed using light microscopy (Eclipse, Ni-E, Nikon) equipped with a 40× magnification optical lens. We further employed a stylus profilometer (Dektak XT, Bruker, USA) to measure the height profile of the particulate deposition structure. Moreover, the FTIR spectra of PAA were recorded using a Nicolet iS50 FTIR spectrometer equipped with a single-reflection diamond-attenuated total reflectance accessory. We measure the zeta potential and size distribution of the particles using a Zetasizer Nano-ZS (Malvern).

### Image Analysis

The average particulate deposition thickness and surface area covered by microparticle deposits were quantitatively analyzed using ImageJ. During our analysis, we converted the image of deposition to binary color form to differentiate between the substrate area covered by particles and between the bare substrate area. We extracted the deposition area on glass by counting image pixels; the pixels were then converted to micrometres using the known scale of the microscope image. The final results of the area covered by deposit and thickness were obtained by averaging over ten different measurements performed on different regions of the microscope image.

## Results and Discussion

**Figure 1:**
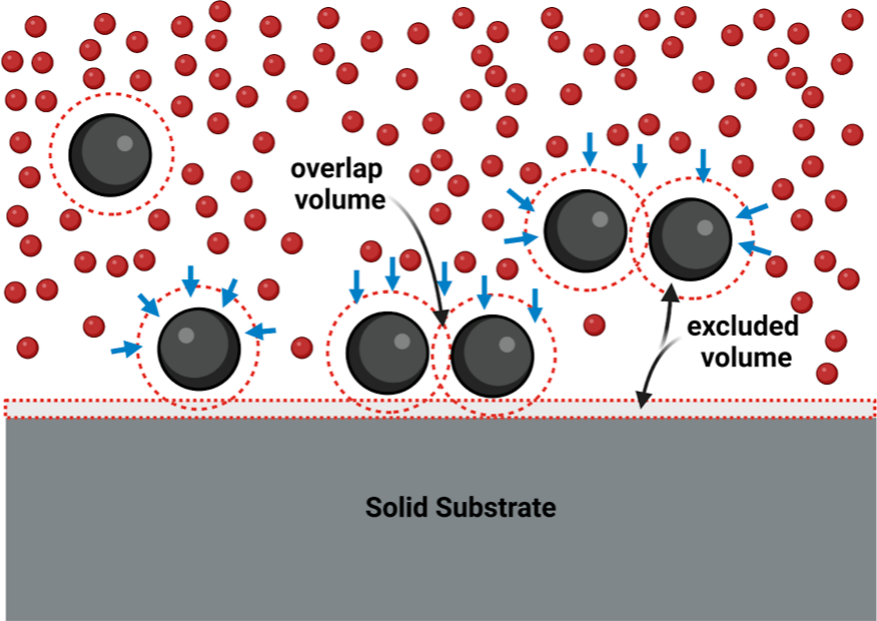
Illustration of attractive depletion of microparticles by nanoparticles on a solid surface. The depletion force between the microparticles and the solid substrate stems from the osmotic pressure gradient in the intervening regions and within the surrounding of the microparticles.

We investigate the contribution of nanoparticles and polymers to the self-assembly of microcolloids on a solid substrate. Nanoparticles induce attractive forces between microcolloids due to osmotic pressure imbalance created by the presence of nanoparticles in the solution.^1,14,54^ This results in a controlled deposition of microcolloids, schematically shown in Figure 1.

**Figure 2:**
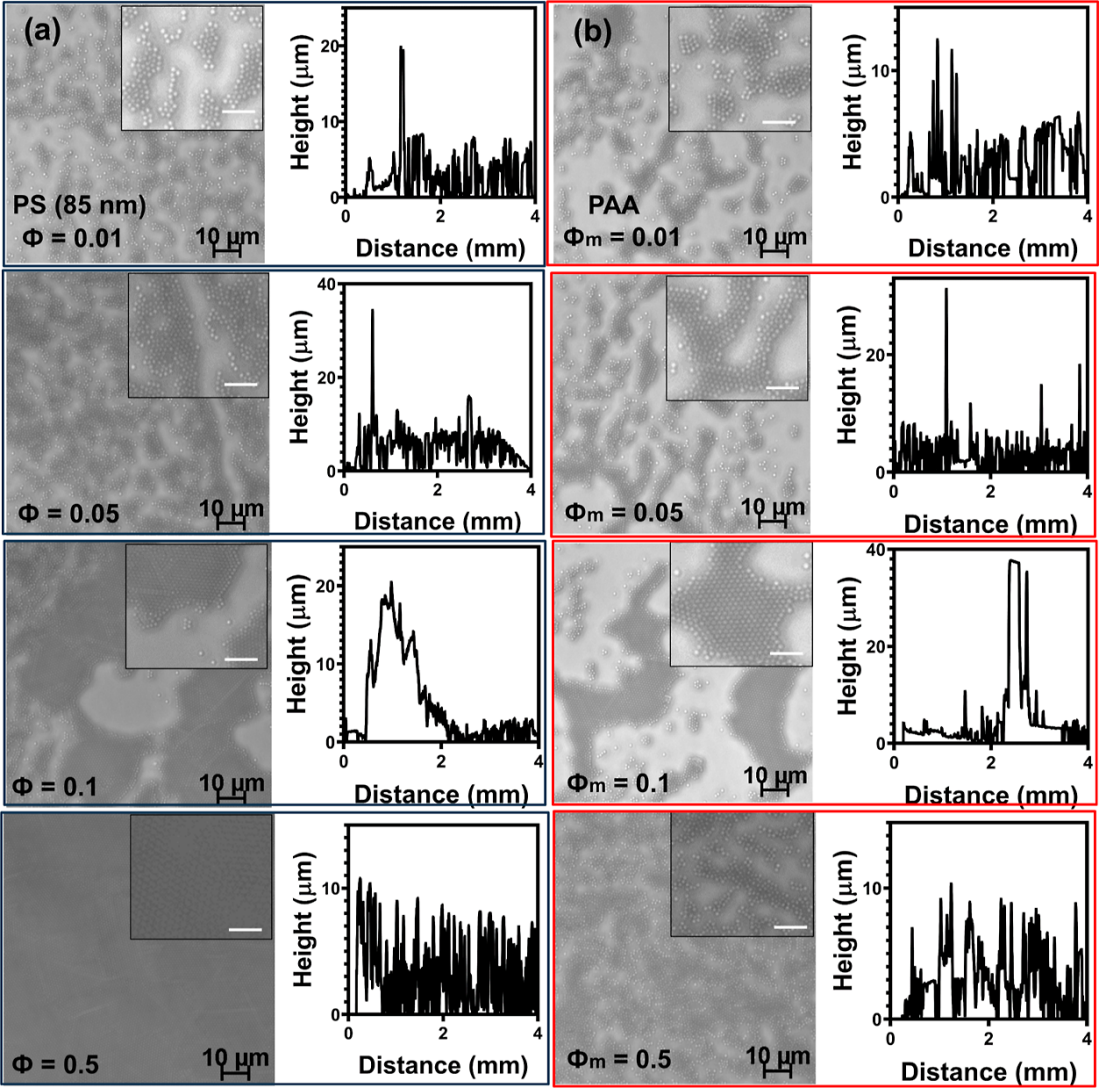
Depletion of 1 μm diameter polystyrene particles at varied concentrations of depletants (a) ϕ = 0.01, 0.05, 0.1 and 0.5% (w/v) PS nanoparticles (85 nm) scan area (100 × 100 μm^2^) (b) ϕ_m_ = 0.01, 0.05, 0.1 and 0.5% (w/v) 100,000 Da. PAA with corresponding profilograms showing the surface roughness of each deposit. Inset shows enlarged images of each assembly with scale bars of 4 μm.

In Figure 2a, we show various morphologies of self-assembled microcolloid structures formed at different concentrations (% w/v) of nanoparticles (ϕ) as depletants. With increasing ϕ, we notice an increase in the surface area covered by microcolloid deposits and in the size of the pattern aggregate structures. This suggests a monotonic increase in the strength of the depletion interaction between microcolloids. We observe a multilayer pattern aggregate structure covering the entire substrate area at ϕ = 0.5% (w/v).

Using profilometry, we further observe that the thickness of the microcolloid aggregate layer adsorbed to the substrate increases with nanoparticle concentration. Nanoparticles as depletants induce the clustering of microcolloids into island-form aggregates at ϕ = 0.01–0.05% (w/v), which eventually coalesce to form large multilayered pattern aggregate structures at ϕ = 0.1–0.5% (w/v). The coalescence of smaller aggregates into larger ones suggests that depletion attraction further enhances aggregate attachments.^55^ We thus observe a clear crossover from flocculation to continuous coating at ϕ = 0.5% (w/v), see Figure 2a. The corresponding profilogram in Figure 2a supports this evidence, showing dense coating of the substrate by large microcolloid aggregates.

Our choice of polymer-depleting agent leads to similar changes in depletion patterns at low to moderate polymer concentrations of ϕ_m_ = 0.01–0.1% (w/v). At ϕ_m_ = 0.5% (w/v), see Figure 2b, we observe a deviation from the case of using nanoparticles, see Figure 2a. The substrate is coated by a fragmented layer of microcolloid aggregates of lesser density than in the case of using nanoparticles as depletion agents. This is likely a result of the adsorption of a fraction of the polymer chains on the microcolloids at high polymer concentrations, yielding simultaneous depletion attraction due to the detached polymer chains and steric stabilization in the presence of the adsorbing polymers.

To check this hypothesis, we conducted zeta potential and particle diameter measurements. We observed a reduction in the zeta potential of the microcolloids from −67.2 to −51.9 mV when the polymer concentration was increased from ϕ_m_ = 0.01 to 0.5% (w/v); see supporting file (Table S1). Dynamic light scattering measurement also confirmed an increase in the diameter of particles in the suspension in this case from the detached microcolloid median size of 1.000 μm to a median size of 1.064 μm, indicating on average a 64 nm layer of adsorbed polymers. Both indications suggest the formation of steric stabilization of the microcolloids at large polymer concentrations, albeit, the coating layer of microcolloids on the substrate is denser than in previous cases of lower polymer concentrations.

The zeta potentials of the various components provide insights into the interparticle and particle–substrate interactions governing microparticle assembly. The glass substrate exhibits a zeta potential level of −54.35 mV, while the PS microparticles and nanoparticles support values of −67.21 and −41.53 mV, respectively, under 5 mM NaNO_3_ ionic conditions; see supporting file (Table S2). Previously explored, the energy barriers arising from surface potentials of this magnitude may stabilize suspended particles against aggregation due solely to double-layer forces.^56,57^ While van der Waals interactions are non-negligible given the submicron particle sizes, they may not dominate the interfacial interactions between particles and particle–substrate.^56^ Rather, considering the prevalence of strong electrostatic repulsions conferred by the zeta potentials, it is most appropriate to attribute the assembly behavior witnessed to the entropically driven depletion forces induced by the crowding of particles and polymer chains.

The assembly mechanism may be the depletion of microcolloids in suspension^55,58^ before deposition on the substrate. This is evidenced by the observation of aggregate deposits on the substrate. However, both phenomena may occur, where preformed aggregates deposit intact, as well as some particles/small clusters continuing to be pushed to the surface from the bulk and assembled following initial contact with the surface and under the influence of cooperative depletion and surface adsorption.^33^

**Figure 3:**
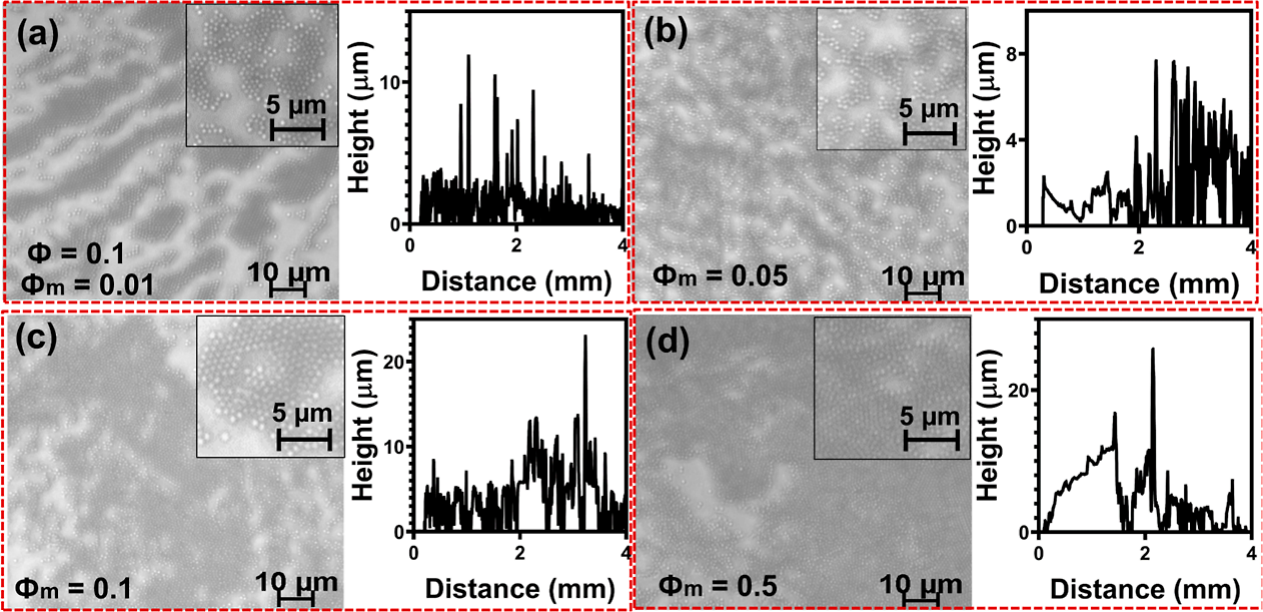
Synergistic depletion of 1 μm diameter polystyrene particles at varied PAA concentrations in ϕ = 0.1% (w/v) PS nanoparticles (85 nm) (a–d) ϕ_m_ = 0.01, 0.05, 0.1 and 0.5% (w/v) PAA, respectively. Scan area (100 × 100 μm^2^). Inset scan area (20 × 20 μm^2^) with scale bars of 5 μm.

We further consider the synergistic effects of nanoparticles and polymers on the assembly of microcolloids on the substrate. Ji and Walz^30^ demonstrated that combining nanoparticles and polymers enhances depletion attraction between suspended microcolloids in the solvent bulk. The particles and polymers in our study partially support this finding in connection to microcolloid deposition on a substrate. We observe that increasing the concentration of PAA [ϕ_m_ = 0.01–0.5% (w/v)] in the presence of our nanoparticles [ϕ = 0.1% (w/v)] results in a monotonic increase in aggregate size on the substrate. The profilogram in Figure 3 at ϕ_m_ = 0.5% (w/v) shows a significant increase in aggregate size atop the substrate. The self-assembly of smaller aggregates into larger structures results in the formation of dense aggregate structures.

The adsorption of polymers on microcolloids reduces the contribution of combined nanoparticle/PAA depletion attraction compared to using just nanoparticles as depletion agents at ϕ = 0.5% (w/v). This is most likely due to the formation of steric barriers between the microcolloids due to the adsorption of PAA on the microcolloid surfaces. Moreover, the substrate coating by microcolloids is denser compared to using only polymers at an overall depletion agent concentration of ϕ_m_ = 0.5% (w/v).

**Figure 4:**
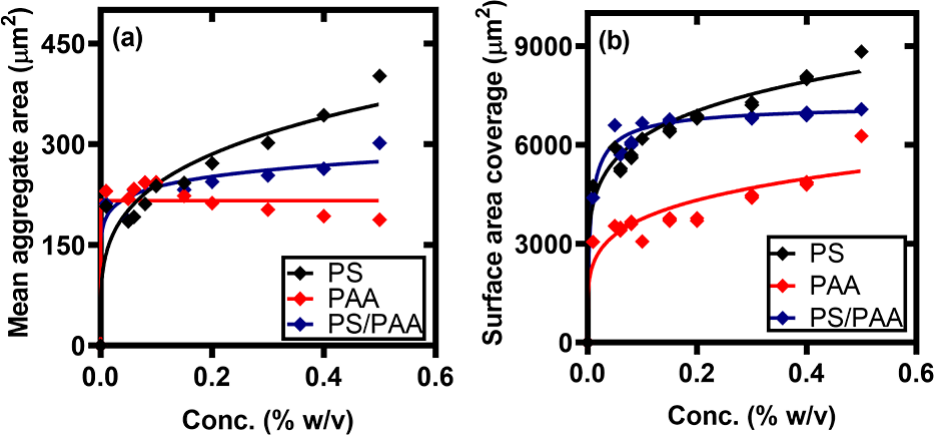
(a) Mean aggregate size of particle deposits on substrates at different depletant concentrations. PS-microcolloid concentration = 0.01% (w/v), (ϕ) = 0.01–0.5% (w/v), (ϕ_m_) = 0.01–0.5% (w/v), while for PS/PAA [(ϕ) = 0.1% (w/v) and (ϕ_m_) = 0.01–0.5% (w/v)]. (b) Surface area covered by microcolloids deposits.

As shown in Figure 4a, the presence of nanoparticles as depletants leads to the formation of larger microcolloid aggregates (400 μm^2^) on the glass substrate compared to aggregates formed with polymer depletants (170 μm^2^) or a mixture of nano- and polymer depletants (280 μm^2^) at 0.5% (w/v) depletant concentrations. This demonstrates that the depletion interaction is stronger for nanoparticles than for polymers. The surface area coverage by the aggregates in Figure 4b follows the same trend, with the maximum coverage obtained with nanoparticle depletants. The mixture of nanoparticles and polymers induced the assembly of aggregates covering more surface area compared to only polymers as depletants.

**Figure 5:**
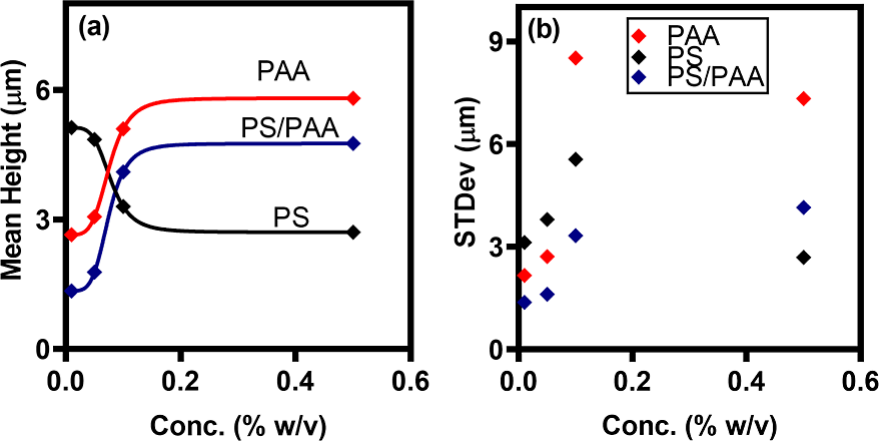
(a) Mean surface roughness of particle deposit on glass substrates under different conditions of depletants obtained from profilograms in Figures 2 and 3. PS-microcolloid concentration = 0.01% (w/v), (ϕ) = 0.01, 0.05, 0.1, and 0.5% (w/v), (ϕ_m_) = 0.01, 0.05, 0.1, and 0.5% (w/v), while for PS/PAA [(ϕ) = 0.1% (w/v) and (ϕ_m_) = 0.01, 0.05, 0.1, and 0.5% (w/v)]. (b) Standard deviation of deposit heights obtained from the profilograms at different concentrations of the depletants.

In Figure 5a, we show a quantitative analysis of the microcolloid deposit roughness using profilometry. The root-mean-square (RMS) roughness gives primary information about the surface topography and variation of effective heights *h*(*x*). We observe a decrease in the deposition RMS roughness with increasing nanoparticle concentration (ϕ) and an increase in the deposition RMS roughness with increasing PAA concentration (ϕ_m_), see Figure 5a. The opposite trends correspond to the uniform and ordered packing of microcolloids with the nanoparticle depletant and less compact packing with the polymer depletant. The trends observed in the root-mean-square (RMS) roughness values (Figure 5) can be attributed to changes in the microstructure of the microparticle deposits with varying depletant conditions. At lower nanoparticle concentrations, island-like formations of microparticle aggregates support uneven deposit topography, characterized by peaks and valleys in the deposit morphology.^59^ As the nanoparticle concentration increases, a transition occurs toward continuous and uniformly packed coating structures on the substrate surface. The emergent homogeneous multilayer morphology possesses a flatter surface profile.

The measured zeta potentials of the polystyrene microcolloids and glass substrate were found to exhibit only minor variation with pH over the 4–9 range investigated, as presented in the Supporting Information (Figure S1). Specifically, the microcolloids displayed zeta potentials that increased moderately from −54 mV at pH 4 to −57 mV at pH 9. The glass substrate followed a similar trend, with values shifting from −56 mV at pH 4 to −61 mV at pH 9. These relatively small changes in surface charge magnitude (−3 and −5 mV for microcolloids and substrate, respectively,) indicate the pH adjustment had negligible influence on the particles’ surface chemistry within the examined conditions. Given double layer theory, such limited fluctuations in zeta potential may not be expected to dramatically impact the interparticle or particle–substrate electrostatic repulsion/attraction balances.^60^ Instead, given the pH-responsiveness of PAA employed as the depletant polymer, it can reasonably be inferred that modifications to PAA’s conformational state and hydrodynamic radius, as regulated by protonation state changes across the tested pH range, may impart dominant influence. Prior studies have characterized PAA’s ionization-dependent coil dimensions and macromolecular properties,^61^ lending credence to its behavior dictating the effective depletion attractions.

**Figure 6:**
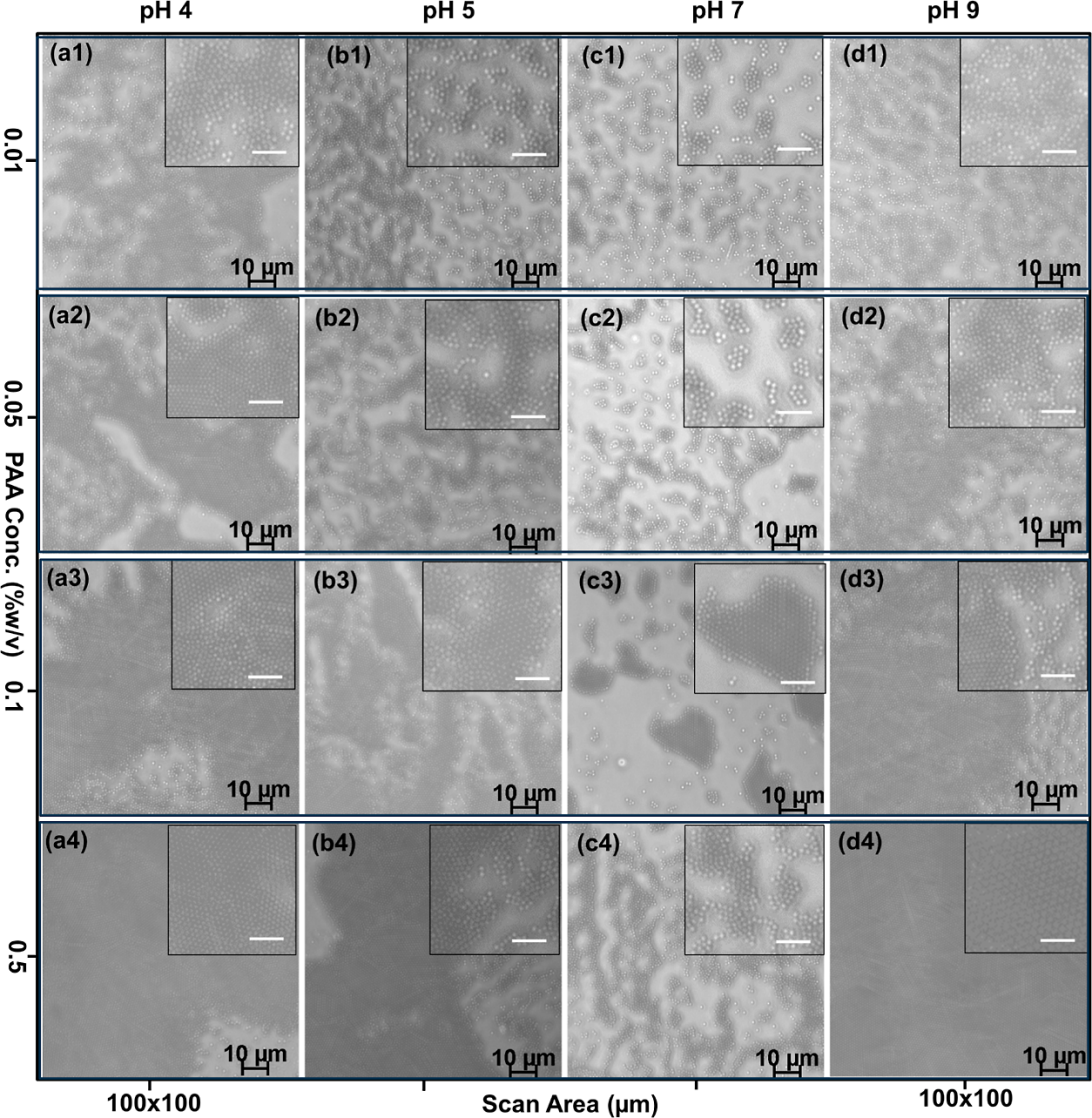
Effect of pH on depletion of 1 μm diameter polystyrene particles at varied PAA concentrations (a) pH 4 [ϕ_m_ = 0.01–0.5% (w/v)] PAA (b) pH 5.25 [ϕ_m_ = 0.01–0.5% (w/v)] PAA (c) pH 7 (ϕ_m_ = 0.01–0.5%) PAA (d) pH 9 [ϕ_m_ = 0.01–0.5% (w/v)] PAA. Scan area (100 × 100 μm^2^). The inset shows enlarged images of each assembly with 4 μm scale bars.

In the present study, we used PAA with a molecular weight of 100,000 Da as a pH-responsive depletant polymer. As reported previously,^61^ PAA has a hydrodynamic radius *R*_H_ that varies from 5.8 nm at pH 4 to 6.6 nm at pH 9. At pH 9, PAA was fully ionized, which increased its charge density, increasing the corresponding electrostatic repulsion between polymer chains, and enhancing depletion attraction forces between microcolloids. This resulted in large, compact aggregates of 420 μm^2^ forming multilayer patterns that covered the largest surface area of 9000 μm^2^, see Figure 6 at ϕ_m_ = 0.5% (w/v).

**Figure 7:**
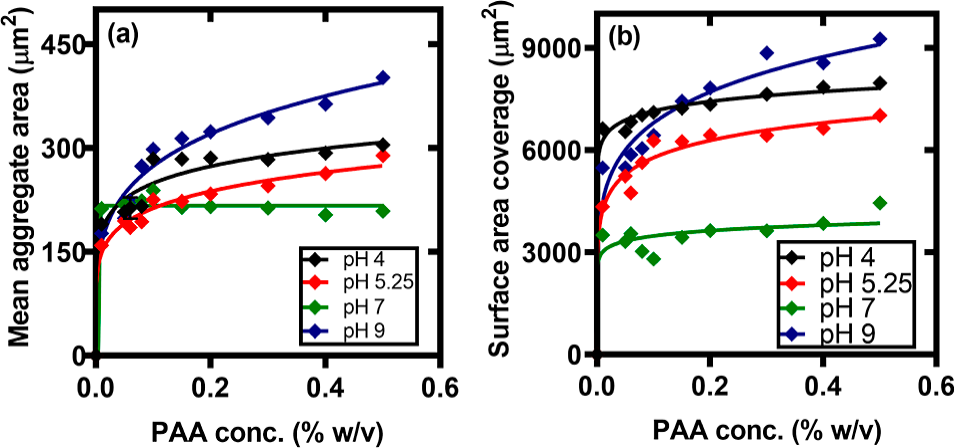
(a) Mean aggregate size of particle deposits on substrates at different depletant concentrations and pH. PS-microcolloid concentration = 0.01% (w/v) and (ϕ_m_) = 0.01–0.5% (w/v). (b) Surface area covered by particles.

The quantitative analysis of aggregation and deposition behavior of microcolloids as shown in Figure 7a,b were highly dependent on suspension pH when poly(acrylic acid) (PAA) was used as a depleting agent. At pH 4 and 7, intermediate aggregate sizes of 300 and 200 μm^2^ were observed with surface coverage of 8000 and 3800 μm^2^, respectively. Visual inspection of the micrographs revealed loosely packed, irregularly shaped clusters with indistinct boundaries. The higher protonation of PAA at pH 4 decreased the radius of gyration of PAA chains, likely favoring their adsorption and bridging between microcolloids and particles/glass substrate to promote flocculation.

**Figure 8:**
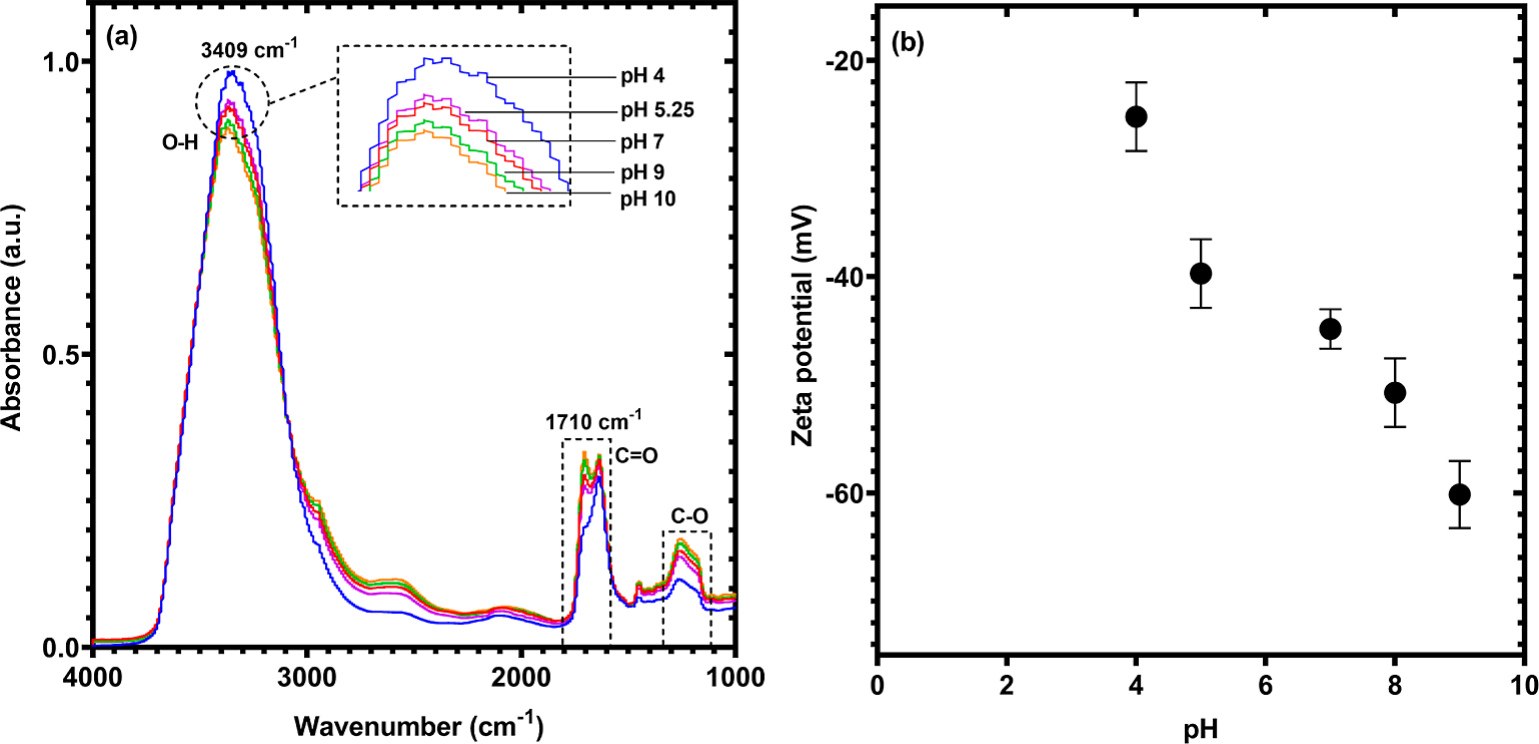
(a) FTIR spectrum of poly acrylic acid PAA at different pH. (b) Zeta potential of PAA in (mV) at different pH.

To further investigate the behavior of PAA at varied pH, we used the Fourier transform infrared spectra of the polymer to check our assertion about polymer ionization states. The pH-dependent shift in the peak positions of the spectra, see Figure 8a, indicates the ionization state and conformational changes of PAA under varying pH conditions. The IR spectra in the O–H and C=O stretching regions show distinct changes in peak positions at varying pH, indicating changes in hydrogen bonding and electrostatic interactions. The intensity of the peak maximum in the O–H stretching region at 3400 cm^–1^ decreases with increasing pH, indicating the dissociation of carboxylic acid groups and increase in the ionization level of PAA. Similarly, in the C=O stretching region at 1707 cm^–1^, we observe a decrease in peak intensity with increasing pH, indicating changes in the conformational state of the polymer chain. We further confirm the ionization and conformational changes of the polymer by measuring pH variations of the zeta potential; see Figure 8b. The results show a significant increase in the negative zeta potential of PAA as pH increases from 4 to 9, indicating an increase in the ionization state of the polymer. Hence, we observe that the ionization and conformational orientations of PAA determine the hydrodynamic radius of the polymer, which in turn affects the depletion forces and self-assembly behavior of microcolloids.

## Conclusions

In summary, we demonstrate the contribution of different depletants, a polystyrene nanoparticle, and a PAA polymer, to the self-assembly behavior of polystyrene microcolloids on a glass substrate. Dip-coating, happy blade, and other methods are used to assemble colloidal particles on substrates. However, we show that particle assembly from dispersed to continuous coating may further occur due to entropic driving forces. We show that depletion forces play a crucial role in varying microcolloid deposition morphology. The nanoparticle as a depletant results in a well-controlled, uniform assembly of particles on glass. However, the polymer as a depletant induces irregular aggregates due to the synergistic effects of depletion and other short-range forces such as steric and bridging^56^ interactions. By tuning the depletion forces through parameters such as depletant concentration and pH, we achieve a range of self-assembled microcolloid patterns on the glass substrate. The synergistic effects of combined polymer and nanoparticle systems enhanced aggregate size and substrate coverage compared to single-polymer component systems. The maximum ordered coating of the substrate was achieved at a concentration of 0.5% (w/v) nanoparticles.

The poly(acrylic acid) ionization state, controlled via pH, strongly influenced aggregate size and surface coverage, giving maximum aggregate size and surface coverage levels at pH 9, where the polymer was fully ionized. FTIR and zeta potential measurements further confirm the ionization of the polymer chain, thereby altering the depletion forces. We present various morphologies of the self-assembled microcolloids, including ordered arrays and disordered aggregates, and demonstrate the versatility of depletion forces in driving microcolloid assembly on a substrate. Our results qualitatively align with model predictions by Minton^33,62,63^ on the behavior of macromolecules, e.g., proteins, in crowded environments. Varying parameters such as depletant types, concentration, and polymer ionization state controlled by pH are direct analogues of influences explored by Minton’s model. Moreover, our work provides experimental validation and extension of theoretical understandings advanced by Minton^33^ and Monterroso et al.^62^ regarding how depletion interactions and external conditions guide macromolecular assembly behaviors in the analysis of proteins and other biochemical molecules. Overall, our findings may be used to manipulate microscale structures into desired deposition patterns using depletion and analyze macromolecules such as proteins subject to their adsorption on surfaces.

## Supporting Information Available

Size distribution of micro and nano polystyrene particles with and without poly(acrylic acid) by DLS measurement; zeta potential of micro and nano polystyrene particles with and without poly acrylic acid by electrophoretic measurement; zeta potential of 1 μm polystyrene particles and glass substrate at different pH values by electrophoretic measurement; characterization data for microcolloids and nanoparticles with 300 × 300 μm^2^ microscopic images of deposits (PDF)
