# Supplementary material for: Depletion-Induced Self-Assembly of Colloidal Particles on a Solid Substrate
Source: Langmuir. 2024 Apr 11;40(16):8554–61. doi: 10.1021/acs.langmuir.4c00186 (PMC11044580; doi:10.1021/acs.langmuir.4c00186)
Supplement: Supplementary file 1 — la4c00186_si_001.pdf [file la4c00186_si_001.pdf]

Depletion-induced self-assembly of colloidal particles on a solid substrate

Gideon Onuh,† Daniel Harries,‡ and Ofer Manor*,†

†The Wolfson Department of Chemical Engineering, Technion – Israel Institute of Technology, Haifa 3200000, Israel

‡The Fritz Haber Research Center, and the Harvey M. Kruger Center for Nanoscience & Nanotechnology, Institute of Chemistry, The Hebrew University, Jerusalem 9190401, Israel

E-mail: manoro@technion.ac.il

Table S1: Zeta potential of micro and nano polystyrene particles with and without polyacrylic acid by electrophoretic measurement

**Particles**	**Zeta potential (mV)**
PS_{micro}	-67.21
PS_{micro} + PAA	-54.93
PS_{nano}	-43.84
PS_{nano} + PAA	-41.53
PAA	-18.31

Table S2: Size distribution of micro and nano polystyrene particles with and without polyacrylic acid by Dynamic Light Scattering (DLS)

**Particles**	**Size (nm)**	**PDI**
PS_{micro}	998.17	0.1761
PS_{micro} + PAA	1000.64	0.2651
PS_{nano}	87.13	0.0287
PS_{nano} + PAA	118.7	0.0174
PAA	6.89	0.3883

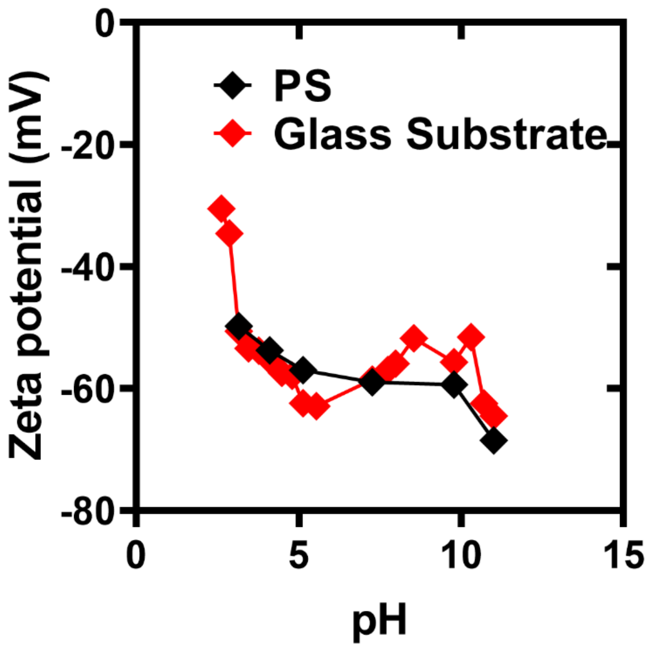

Figure S1: Zeta potential of 1 μm polystyrene particles and glass substrate at different pH values by electrophoretic measurement.

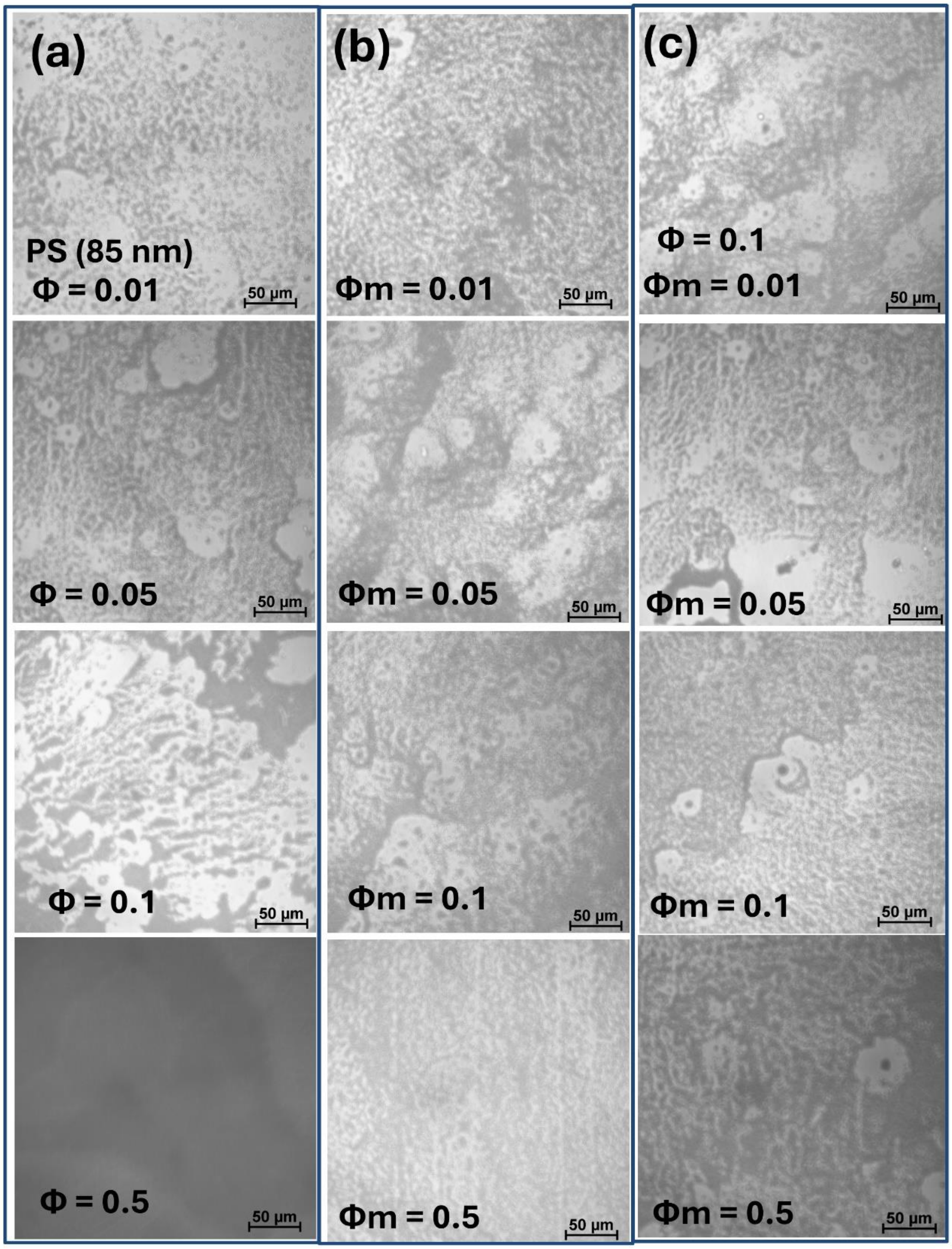

Figure S2: Depletion of 1 μm diameter polystyrene particles at varied concentrations of depletants (a) ϕ = 0.01, 0.05, 0.1 and 0.5 % PS nanoparticles (85 nm) scan area (300x300 μm2) (b) ϕ_m = 0.01, 0.05, 0.1 and 0.5 % 100,000 Dalt. PAA (c) Mixture of nanoparticles and polymers as depletant ϕ = 0.1 % nanoparticles and ϕ_m = 0.01, 0.05, 0.1, and 0.5 % PAA.
